# Supplementary material for: Ag3PO4-Deposited TiO2@Ti3C2 Petals for Highly Efficient Photodecomposition of Various Organic Dyes under Solar Light
Source: Nanomaterials (Basel). 2022 Jul 18;12(14):2464. doi: 10.3390/nano12142464 (PMC9322657; doi:10.3390/nano12142464)
Supplement: Supplementary file 1 [file nanomaterials-12-02464-s001.zip › nanomaterials-1776022-supplementary.pdf]

nanomaterials

Supplementary Information for:

Ag_3PO_4-Deposited TiO_2@Ti_3C_2 Petals for Highly Efficient Photodecomposition of Various Organic Dyes under Solar Light

Ngoc Tuyet Anh Nguyen and Hansang Kim *

Department of Mechanical Engineering, Gachon University, Seongnam 13120, Korea; anh2510@gachon.ac.kr
* Correspondence: hskim70@gachon.ac.kr; Tel.: +82-31-750-5307

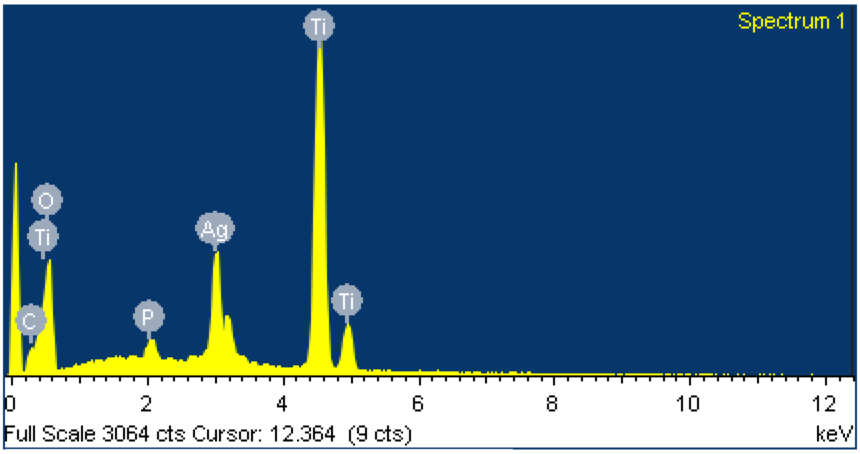

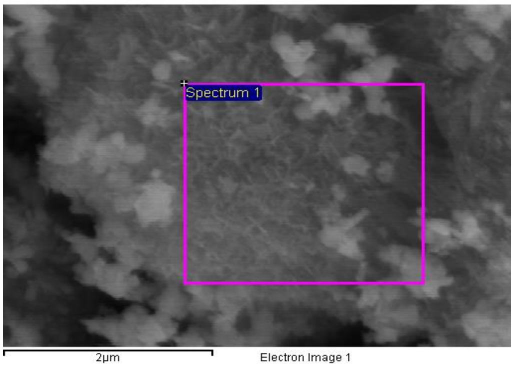

Element	Atomic%
C K	6.61
O K	62.10
P K	0.89
Ti K	26.21
Ag L	4.18

Figure S1. EDS patterns of Ag_3PO_4/TiO_2@Ti_3C_2 (sample A4); the inset image shows a SEM image of the sample.

Citation: Nguyen, N.T.A.; Kim, H. Ag_3PO_4-Deposited TiO_2@Ti_3C_2 Petals for Highly Efficient Photodecomposition of Various Organic Dyes under Solar Light. *Nanomaterials* **2022**, *12*, 2464. https://doi.org/10.3390/nano12142464

Academic Editors: Junying Zhang, Yong Chen and Jungang Hou

Received: 1 June 2022
Accepted: 9 July 2022
Published: 18 July 2022

Publisher's Note: MDPI stays neutral with regard to jurisdictional claims in published maps and institutional affiliations.

Copyright: © 2022 by the authors. Submitted for possible open access publication under the terms and conditions of the Creative Commons Attribution (CC BY) license (https://creativecommons.org/licenses/by/4.0/).

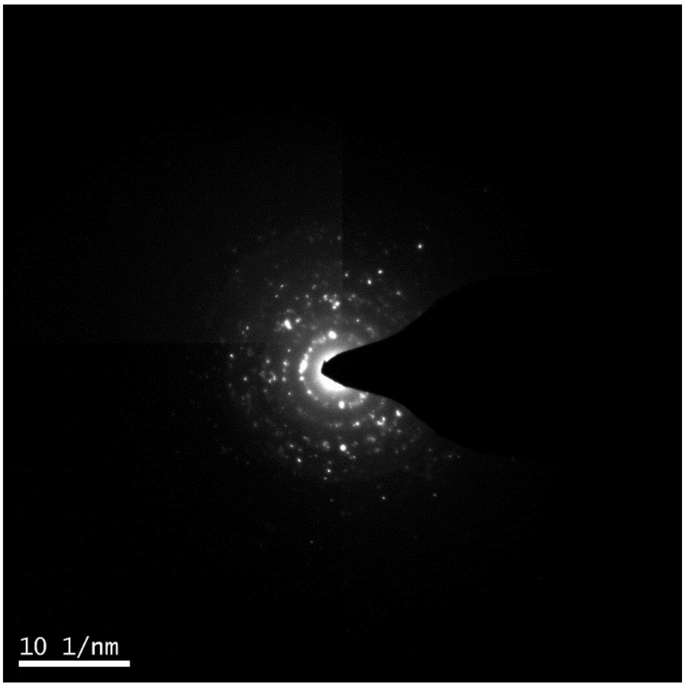

Figure S2. SAED image of $Ag_3PO_4/TiO_2@Ti_3C_2$ (sample A4) taken of an arbitrary area.

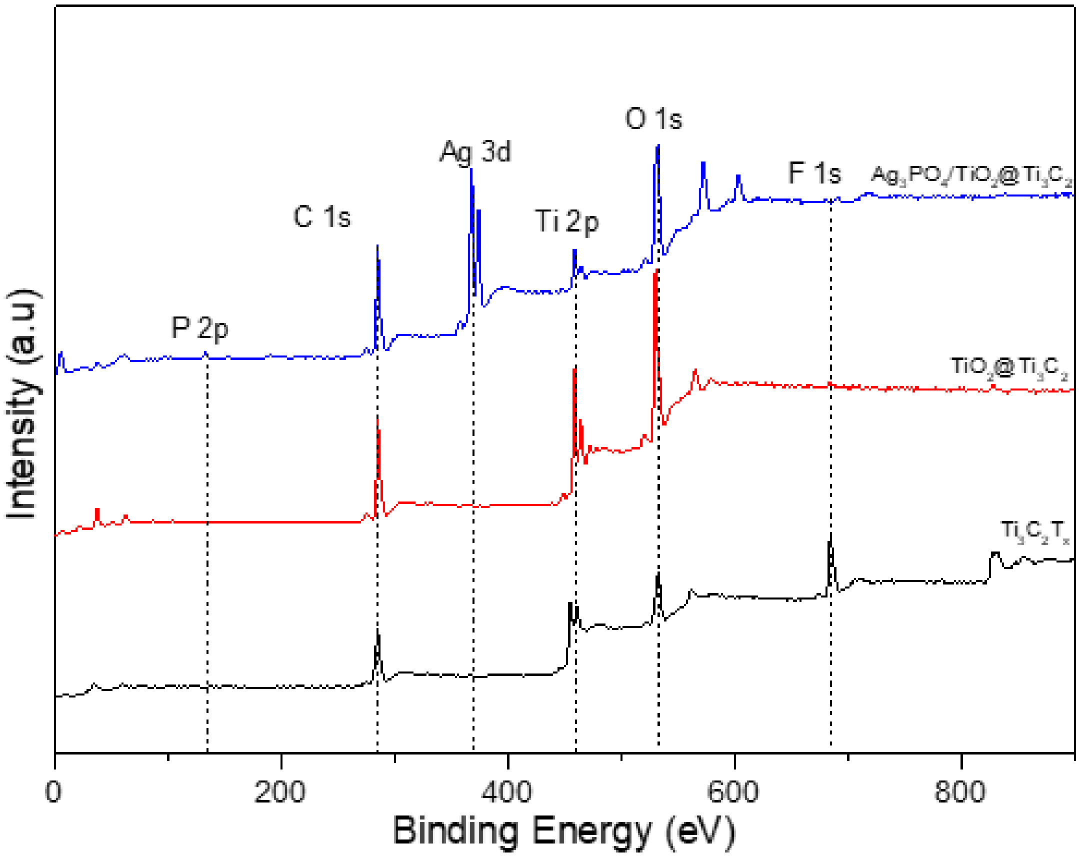

Figure S3. XPS survey spectra of Ti_3C_2, $TiO_2@Ti_3C_2$ and $Ag_3PO_4/TiO_2@Ti_3C_2$.

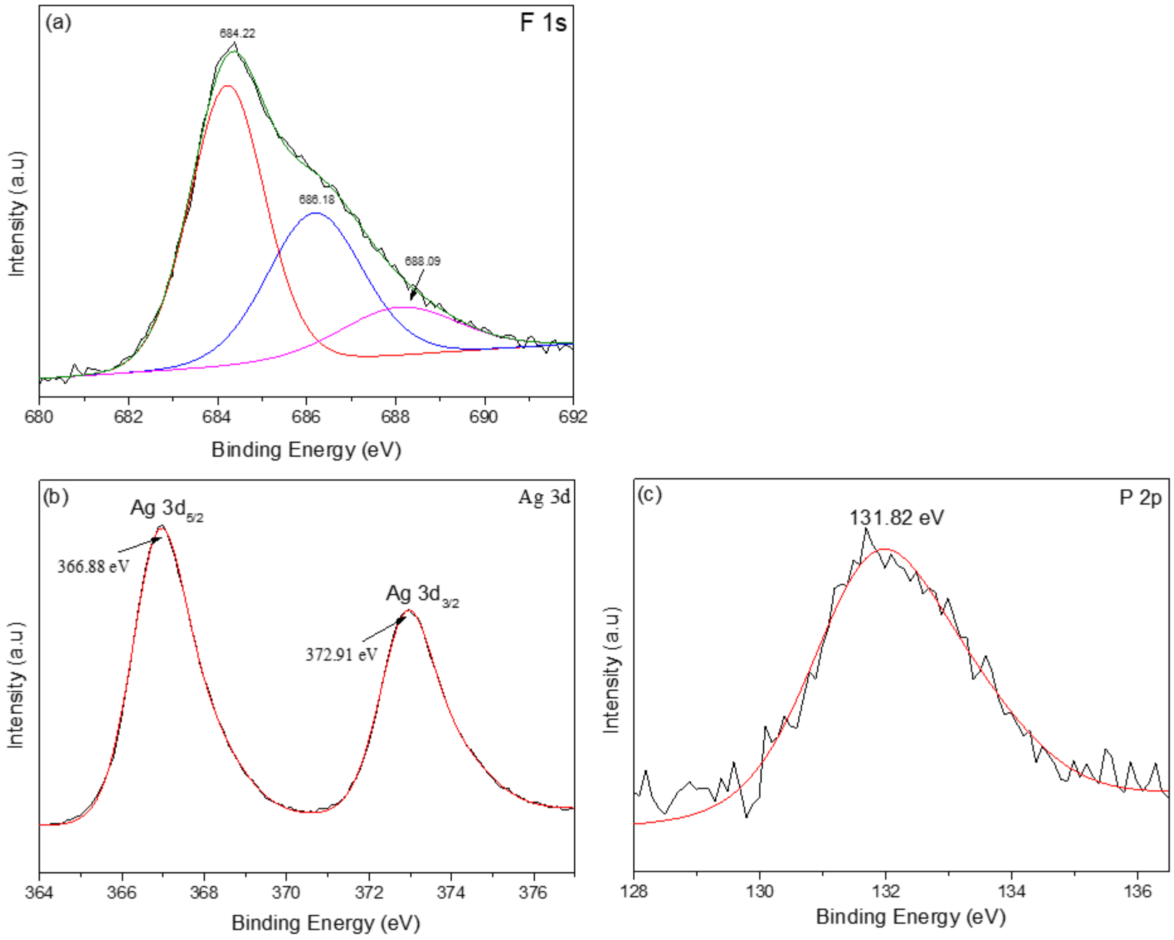

Figure S4. (a) F 1s high-resolution XPS spectra of Ti_3C_2, and (b-c) Ag 3d and P 1s high-resolution XPS spectra of $Ag_3PO_4/TiO_2@Ti_3C_2$ (sample A4).

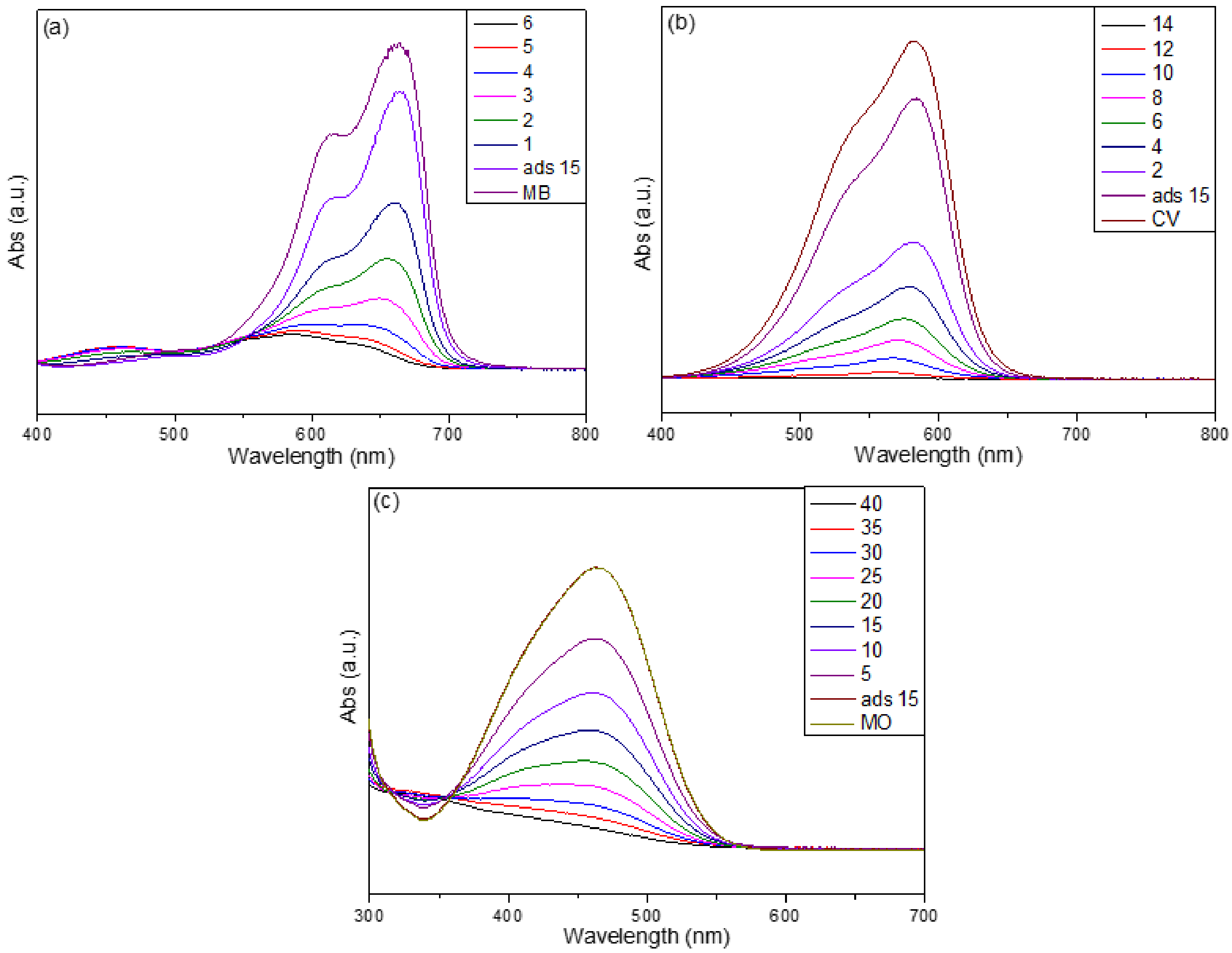

Figure S5. Photodegradation absorption spectra of (a) Methylene Blue, (b) Crystal Violet, (c) Methylene Orange under solar light.
